# Phosphorus Chemistry at the Roots of Bioenergetics: Ligand Permutation as the Molecular Basis of the Mechanism of ATP Synthesis/Hydrolysis by F_O_F_1_-ATP Synthase

**DOI:** 10.3390/molecules28227486

**Published:** 2023-11-08

**Authors:** Sunil Nath

**Affiliations:** 1 Department of Biochemical Engineering and Biotechnology, Indian Institute of Technology Delhi, Hauz Khas, New Delhi 110016, India; sunath@iitd.ac.in or sunil_nath_iit@yahoo.com; 2 Institute of Molecular Psychiatry, Rheinische-Friedrichs-Wilhelm Universität Bonn, D-53127 Bonn, Germany

**Keywords:** rotary ATPases, F_O_F_1_/F_1_-ATPase, ATP synthase, ATP synthesis and ATP hydrolysis, molecular mechanism, kinetics and catalysis, oxygen exchange, phosphorus chemistry and biochemistry, ligand permutation and ligand displacement/substitution, Boyer’s binding change mechanism of ATP synthesis/hydrolysis, Nath’s torsional mechanism of ATP synthesis/hydrolysis, two-site vs. three-site models of ATP synthesis/hydrolysis, bisite catalysis vs. trisite catalysis

## Abstract

The integration of phosphorus chemistry with the mechanism of ATP synthesis/hydrolysis requires dynamical information during ATP turnover and catalysis. Oxygen exchange reactions occurring at β-catalytic sites of the F_O_F_1_-ATP synthase/F_1_-ATPase imprint a unique record of molecular events during the catalytic cycle of ATP synthesis/hydrolysis. They have been shown to provide valuable time-resolved information on enzyme catalysis during ATP synthesis and ATP hydrolysis. The present work conducts new experiments on oxygen exchange catalyzed by submitochondrial particles designed to (i) measure the relative rates of Pi–ATP, Pi–HOH, and ATP–HOH isotope exchanges; (ii) probe the effect of ADP removal on the extent of inhibition of the exchanges, and (iii) test their uncoupler sensitivity/resistance. The objectives have been realized based on new experiments on submitochondrial particles, which show that both the Pi–HOH and ATP–HOH exchanges occur at a considerably higher rate relative to the Pi–ATP exchange, an observation that cannot be explained by previous mechanisms. A unifying explanation of the kinetic data that rationalizes these observations is given. The experimental results in (ii) show that ADP removal does not inhibit the intermediate Pi–HOH exchange when ATP and submitochondrial particles are incubated, and that the nucleotide requirement of the intermediate Pi–HOH exchange is adequately met by ATP, but not by ADP. These results contradicts the central postulate in Boyer’s binding change mechanism of reversible catalysis at a F_1_ catalytic site with $K_{eq}\sim 1$ that predicts an absolute requirement of ADP for the occurrence of the Pi–HOH exchange. The prominent intermediate Pi–HOH exchange occurring under hydrolytic conditions is shown to be best explained by Nath’s torsional mechanism of energy transduction and ATP synthesis/hydrolysis, which postulates an essentially irreversible cleavage of ATP by mitochondria/particles, independent from a reversible formation of ATP from ADP and Pi. The explanation within the torsional mechanism is also shown to rationalize the relative insensitivity of the intermediate Pi–HOH exchange to uncouplers observed in the experiments in (iii) compared to the Pi–ATP and ATP–HOH exchanges. This is shown to lead to new concepts and perspectives based on ligand displacement/substitution and ligand permutation for the elucidation of the oxygen exchange reactions within the framework of fundamental phosphorus chemistry. Fast mechanisms that realize the rotation/twist, tilt, permutation and switch of ligands, as well as inversion at the γ-phosphorus synchronously and simultaneously and in a concerted manner, have been proposed, and their stereochemical consequences have been analyzed. These considerations take us beyond the binding change mechanism of ATP synthesis/hydrolysis in bioenergetics.

## 1. Introduction

The central importance of phosphorus (P)-containing compounds has a long and fabled history, and their wide usage as reagents, precursor molecules, catalysts and solvents in the laboratory has been carefully documented in the literature [1,2,3,4]. The application of the P-compounds in industry and medicine has also been well described [5,6,7]. Phosphorus constitutes a key essential component of all forms of life and many important biomolecules, such as nucleic acids, phospholipids, and adenosine triphosphate (ATP), the universal biological energy currency. If “the golden age of phosphorus chemistry” that we are currently living in is to be fully realized, progress on the biochemical aspects of phosphorus chemistry needs to go hand-in-hand with the rapid advances made in the synthesis and isolation of the P-compounds [1,2,3,4,5,6,7].

Despite longstanding interest in the biological properties of the P compounds, including ATP [5,6,7,8,9], progress in phosphorus biochemistry has lagged behind. To a large extent this has to do with the fact that it is not sufficient to study the properties of ATP in solution using standard chemical techniques. One needs to at least include in the analysis the large ~650,000 Da enzyme and rotary transducer of the ATP synthase that synthesizes the ATP, or better still the mitochondrial organelle or its physiologically functional sub-system in submitochondrial particles, thereby making it a problem of complex systems. The problem of ATP molecular mechanism and enzyme dynamics has continued to occupy the central stage, since bioenergetics and metabolism are fundamentally important in both cell life as well as in health and disease. To improve our understanding of the energy dynamics of rotary biomolecular machines, various physical [10], chemical [11], biological [12], mathematical [13], and energy landscape approaches [14,15] have been developed and applied to the ATPases. Rotary and linear molecular motors have been studied both experimentally [16,17,18,19] and computationally [20,21,22], and molecular dynamics [20] and multiscale simulations [21,22] have been performed. Thermodynamic [23,24,25,26,27], electrostatic [28,29,30] and molecular systems biology/engineering [31,32,33] approaches have also been developed to understand the function of these complex nanosystems. The developments in ATP and cell life [33,34,35,36,37,38] and cell death [39,40,41,42,43,44,45,46,47] have been reviewed previously.

However, the integration of the mechanism of ATP synthesis/hydrolysis with phosphorus chemistry requires biochemical information on the dynamical characteristics of phosphorus–oxygen ligands of an ATP molecule in the active site of the ATP synthase/F_1_-ATPase enzyme during catalysis. How can such time-resolved information on elementary events occurring within very small systems of the single catalytic sites of enzymes, such as in the β-subunits of the F_O_F_1_-ATP synthase/F_1_-ATPase, be gathered and interpreted? This is a formidable problem. Although time-resolved techniques have continued to be improved and innovated [48,49,50,51], their applications to large multi-subunit enzymes and organized assemblies of molecules such as the F_1_-ATPase/ATP synthasome or respiratory supercomplexes are scarce. A promising experimental approach that directly monitored rotation in ATP synthase in real time by individual F_1_F_O_ molecules used confocal single-molecule FRET techniques with freely diffusing proteoliposomes. The technique was further extended in a pioneering three-fluorophore experiment that could successfully identify mechanical deformations in the rotor subunits [52]. Problems of limited time resolution continue to be addressed. However, the F_1_-ATPase dynamics are incompletely understood and continue to be addressed by biochemical [53], biophysical [52,54], and systems biology [31,55] approaches.

**Figure 1:**
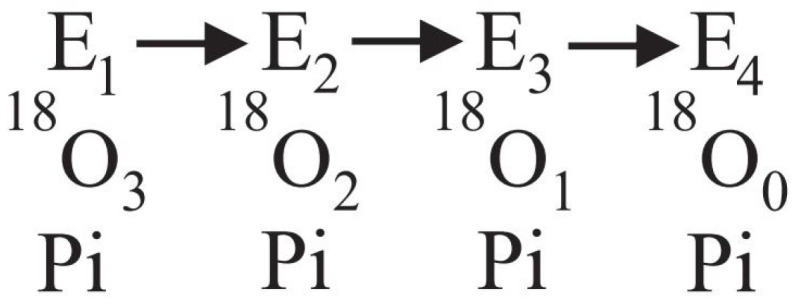
Schematic of the oxygen exchange reactions within a single β-catalytic site in the F_1_ portion of the F_O_F_1_-ATP synthase/F_1_-ATPase. The process starts from an initial state of the system with a known distribution of the ^18^O label in the γ-phosphoryl group of ATP (E_0_; not shown in the schematic). Following the terminal bond cleavage of the bound ATP and the release of inorganic phosphate (Pi), the system progresses between enzyme states E_1_, E_2_, E_3_, and E_4_ containing, respectively, 3, 2, 1, and 0 atoms of ^18^O in the released Pi molecule due to the intermediate Pi–HOH exchange occurring during ATP hydrolysis, as shown by arrows. The ^18^O_3_, ^18^O_2_, ^18^O_1_, and ^18^O_0_ Pi distributions are measured, and the estimated distributions obtained by a stochastic kinetic theory [57] are compared with the experimental distributions.

An interesting methodology by which time-resolved information on catalytic site events can be collated is by monitoring the oxygen exchange reactions [56,57,58]. As is well known, experimental data on the oxygen exchange reactions in the ATP synthesis mode by the F_O_F_1_-ATP synthase are scarce [57]. In particular, the characteristics of the intermediate ATP–HOH exchange, considered as a diagnostic of the oxidative phosphorylation process, was experimentally studied by Nath and coworkers [57]. A stochastic kinetic theory was developed from first principles to support the experiments, and to analyze and interpret experimental data in the synthesis and hydrolysis modes. The variables in the concatenated system of differential equations were solved sequentially and shown to lead to a Poisson process as the steady state solution of the continuous-time Markov chain [57]. However, given that the vast majority of studies have focused on the hydrolysis mode, very good data on the exchange reactions occurring during ATP hydrolysis by F_1_-ATPase recorded by other workers, notably on the intermediate Pi–HOH exchange (Figure 1), already exist in the literature [58].

The above works have stimulated the development of at least two different theories or mechanisms of ATP synthesis/hydrolysis. Boyer’s binding change mechanism [59,60,61] was primarily arrived at based on the action of uncouplers on the exchange reactions [59]. It postulated reversible catalysis and site–site cooperativity, and proposed that external energy input is primarily required for the release of bound ATP from a catalytic site, but not for its synthesis [59,60,61]. An alternative Nath’s torsional mechanism of energy transduction and ATP synthesis/hydrolysis [18,30,31,32,33,57,62,63,64] considers an irreversible mode of catalysis and asymmetric interactions of the catalytic sites with the single-copy γ- and ε-subunits of F_1_, and proposes that every elementary step in ATP synthesis requires energy [65,66,67,68,69,70,71,72,73,74]. The results presented here are consistent with oxygen exchange occurring at all three catalytic sites during the process of hydrolysis by F_1_-ATPase at high ATP concentrations.

In the experiments where ^18^O water is used, and since an oxygen from Pi forms water during ATP synthesis [75], one oxygen atom from medium water gets incorporated into the Pi released during the reverse ATP hydrolysis process (Equation (1)). Hence the minimum value of ^18^O/P—that corresponds to zero exchange, and occurs at ≥3 mM ATP concentrations—is 1, and the maximum is 4 (i.e., on average, all four oxygen atoms in a released Pi molecule are labeled with four ^18^O from water). Hence the values of ^18^O/P recorded in these experiments vary between 1 and 4.

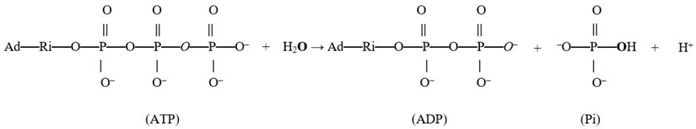
(1)

The results of new experiments on submitochondrial particles described in Section 3 are presented in Section 2.1. These include new results on the relative rates of the three isotope exchanges, studies on the inhibition of these exchanges upon ADP removal in the presence of an ATP regenerating system, and reports on uncoupler sensitivity/resistance of the exchanges. Their myriad biological implications are discussed in Section 2.2.1, Section 2.2.2, Section 2.2.3 and Section 2.2.4. New concepts based on ligand permutation have been developed to explain oxygen exchange in oxidative phosphorylation in Section 2.2.5. These concepts are integrated into fundamental phosphorus chemistry in Section 2.2.5; a concerted mechanism with inversion at the phosphorus center is proposed, and its stereochemical consequences are analyzed in Section 2.2.6. Section 3 describes the experimental methods and Section 4 presents the main conclusions of the study.

## 2. Results and Discussion

### 2.1. Novel Insights from Oxygen Exchange Experiments on Submitochondrial Particles

Fifty years ago, based on his interpretation of ^18^O exchange experiments, Boyer proposed the hypothesis that there exists a catalytic site on F_1_ that performs reversible ATP synthesis/hydrolysis with $K_{eq}\sim 1$ during steady-state functioning at physiological rates [59]. For hydrolysis at high ATP, a true reaction equilibrium constant cannot be measured by ^18^O exchange methodology. As a matter of fact, it was indeed acknowledged ten years later that “measurement of the equilibrium constant for the reversible hydrolysis of bound ATP at high ATP concentrations would be useful but does not appear to be readily accessible experimentally” [58]. Where does this leave us today vis-à-vis this and other associated dogma?

The above dogma/concept has been criticized by several workers [31,33,34,76,77,78,79,80]. For example, under one concept, an F_1_-ATPase catalytic site with $K_{eq}\sim 1$ generates ATP and ADP in a ratio close to unity, and this has the consequence that a binding change leads to the release of ATP only half the time. Any mechanism that shifts the equilibrium in the site towards ATP requires external energy input, thereby falsifying the proposal of the binding change mechanism.

As a result of these difficulties, we started to re-assess the fundamentals of the problem from 25 years ago [62,63,64]. We were challenged to formulate a new molecular theory of ATP synthesis/hydrolysis that could “explain the wealth of existing data and better withstand further experimental challenge” [33,77,78]. At the roots of Boyer’s binding change mechanism lay the hypothesis that the various exchange reactions accompanying oxidative phosphorylation and photophosphorylation are explained by a dynamic reversal of ATP formation at an F_1_-catalytic site [59]. In other words, the mechanism postulates that the overall reaction moves back and forth between ATP synthesis and hydrolysis at the catalytic site. It was estimated from oxygen exchange studies that, at low ATP concentrations (<1 µM) in the hydrolysis mode, interconversion between substrates and products at an F_1_-ATPase catalytic site occurred >400 times before product Pi release [58]. Based on such estimates and the relative uncoupler resistance of the Pi–HOH exchange, compared to the inhibition of the Pi–ATP and ATP–HOH exchanges by uncouplers, it was proposed for the ATP synthesis mode that [59,60]:

(i). The energy of the ion gradients is required primarily to induce a conformational change that enables product ATP release from an F_1_ catalytic site;
(ii). ATP synthesis occurs at a second catalytic site in F_1_ without external energy input.

The binding change mechanism would predict an absolute requirement of ADP for the occurrence of the Pi–HOH exchange. From ^18^O exchange experiments on bovine heart submitochondrial particles, Boyer and coworkers had indeed suggested the involvement of ADP as a substrate in the reversible phosphorylation reaction, and proposed this as the basis for the apparent absolute requirement for ADP to catalyze a prominent Pi–HOH exchange [81].

**Figure 2:**
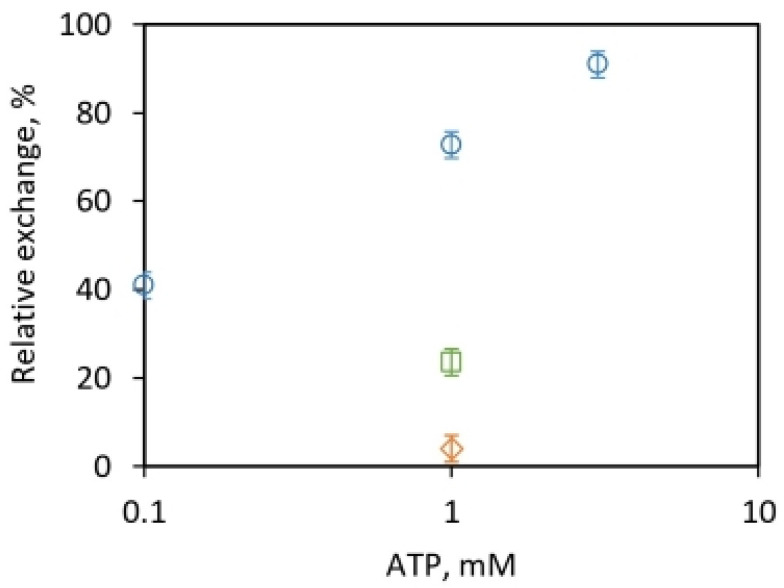
Effect of ADP removal on the Pi–ATP, Pi–HOH and ATP–HOH isotope exchanges at 5 mM Mg^2+^ and varying concentrations of ATP added to submitochondrial particles in 15 min incubations in the reaction medium given in Section 3 (mean ± SD, *n* = 6). The percentage of maximal extent after normalization is plotted on the ordinate. The maximum value (control) for each isotope exchange was taken as the number of atoms exchanged in the absence of the addition of pyruvate kinase. Error bars lie within the symbol size selected to represent the data. The medium water contained 0.7 atom% excess of ^18^O. The open gold diamond (◇) shows the almost complete depression of the Pi–ATP exchange upon ADP removal by addition of phosphoenolpyruvate and excess pyruvate kinase; the open green square (□) represents the markedly inhibited ATP–HOH exchange, and open blue circles (○) show the robust intermediate Pi–HOH exchange that continues prominently on ADP removal.

We explored innovative ways of using the isotope exchanges to test the above concepts and address longstanding serious shortcomings. Submitochondrial particles constituted the ideal model system to test for adenine nucleotide requirements of the exchanges because they lack the nucleotide pools of isolated mitochondria. The results shown in Figure 2 verify the dependence of the intermediate Pi–HOH exchange on the adenine nucleotides. However, they reveal the important fact that this obligatory nucleotide requirement for the Pi–HOH exchange is met by ATP, and not by ADP.

The results of Figure 2 show that even when ADP is depleted to a low level by the addition of excess pyruvate kinase and phosphoenolpyruvate, the intermediate Pi–HOH exchange is only inhibited slightly (by <25% or <10% at 1 mM and 3 mM ATP, respectively)—5 mM Mg^2+^ compared to control (without the addition)—and this continues to occur in a prominent way. On the other hand, ADP removal markedly depresses the Pi–ATP and ATP–HOH exchanges—by 96% and 77%, respectively, at 1 mM ATP and 5 mM Mg^2+^ (Figure 2). Similar results on the dependence on ADP are obtained for submitochondrial particles of varying net phosphorylation capacities between 10 and 15 µmol ATP mg^−1^ h^−1^. Similar trends were seen as long as Mg^2+^ concentrations were maintained higher than ATP concentrations.

The data shown in Figure 2 yield relative rates of the Pi–ATP, ATP–HOH, and Pi–HOH exchanges of 1:6:18 at 1 mM ATP and 5 mM Mg^2+^ in long-time (15 min) incubations with submitochondrial particles. These are similar to the observed relative rates of the exchanges in the absence of uncouplers or pyruvate kinase addition in mitochondria [59]. However, in the short-time incubations (90 s incubations with reduced ATP turnover that ensures the maintenance of very high ATP to ADP concentrations) at otherwise the same conditions of 1 mM ATP and 5 mM Mg^2+^, as in Figure 2, these ratios were increased to 1:16:48 in the presence of pyruvate kinase and phosphoenolpyruvate.

The above experiments show that under conditions of ADP depletion by the addition of pyruvate kinase and phosphoenolpyruvate to submitochondrial particles, relative rates of the Pi–ATP, ATP–HOH, and Pi–HOH exchanges of 1:16:48 are obtained. Thus, ADP removal leads to conditions for the continued presence of high intermediate Pi–HOH exchange along with a marked inhibition of both the Pi–ATP and ATP–HOH exchanges. This has major biological implications that shall be discussed in Section 2.2.

From the experiments, the strongest, most compelling piece of evidence we can provide that the intermediate Pi–HOH exchange does not require the dynamic reversal of ATP formation from ADP and Pi, as per a central tenet of Boyer’s binding change mechanism, is the inability of excess pyruvate kinase and phosphoenolpyruvate, and thereby of ADP removal, to inhibit the Pi–HOH exchange when ATP and submitochondrial particles are added in long-time incubations in medium water with 0.7 atom% excess ^18^O (Figure 2) and short-time incubations with 10 atom% excess ^18^O in medium H_2_O (discussed above). The continued occurrence of an appreciable Pi–HOH exchange under these conditions is best explained as one caused by and accompanying the essentially irreversible cleavage of ATP by submitochondrial particles—as proposed by Nath’s torsional mechanism of energy transduction and ATP synthesis/hydrolysis—independent from a reversible formation of ATP from ADP and Pi. ATP (but not ADP) is sufficient to meet the nucleotide requirement of this essentially irreversible [33] ATP hydrolysis process [31,77,78].

**Figure 3:**
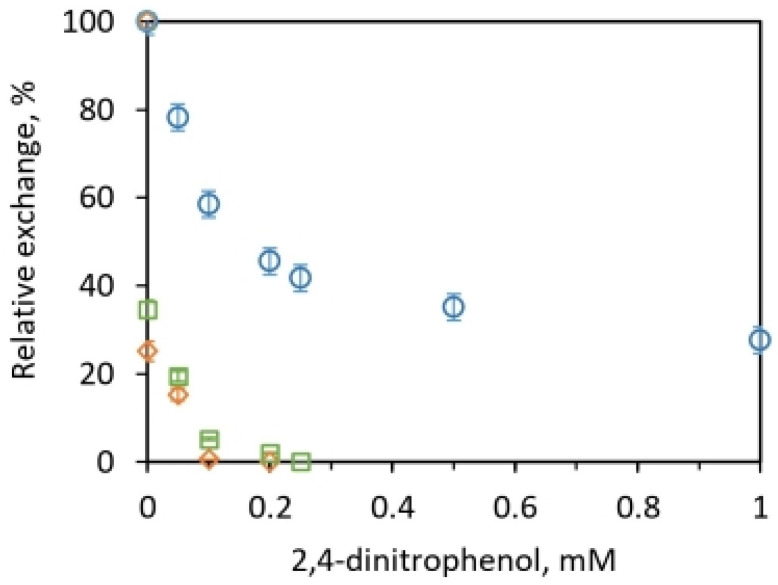
Dependence of the exchange reactions catalyzed by submitochondrial particles under the conditions given in Section 3 on the concentration of the weak anionic uncoupler of oxidative phosphorylation 2,4-dinitrophenol (mean  ±  SD, *n* = 6). Open gold diamonds (◇) represent the Pi–ATP exchange, open green squares (□) show the ATP–HOH exchange, while open blue circles (○) show the continued, relatively uncoupler-resistant Pi–HOH exchange.

In fact, our biochemical results on uncoupling shown in Figure 3 reveal that the intermediate exchange reactions in mitochondrial systems themselves can be divided into two categories. One category consists of the Pi–ATP and ATP–HOH exchanges that require ADP and Pi, are sensitive to uncouplers, and are closely associated with oxidative phosphorylation capacity. A second category comprises the intermediate Pi–HOH exchange identified above that is associated with ATP hydrolysis and is relatively far more insensitive to the uncoupling action of the classical uncouplers of oxidative phosphorylation [59], as shown by the results in Figure 3. These results and other biochemical implications are further discussed in detail in Section 2.2.4.

### 2.2. Biological Implications of the Results of Isotope Exchange Experiments on Submitochondrial Particles

We are now in a position to discuss the development of concepts, integrate the various aspects of the exchange reactions found in this work, and consider the various biological implications arising.

We started by considering the overall phosphorylation chemistry of oxidative and photosynthetic phosphorylation, which is summarized by the deceptively simple Equation (1). Specifically, it shows that an oxygen atom from Pi forms water, and that it is an oxygen atom that is part of the β-phosphate group of ADP that engages in a covalent P–O bond with the phosphorus atom of substrate Pi. However, as discussed above, the ATP synthase not only catalyzes the synthesis and hydrolysis of ATP (Equation (1)) but also an exchange of ^18^O from H^18^OH into the γ-phosphate P center of ATP (ATP–HOH exchange), and also into inorganic phosphate (Pi–HOH exchange). Unlike the Pi–ATP isotopic exchange that occurs at rates approximately equal to the rate of ATP synthesis, the rates of the oxygen exchanges can be rapid relative to the rate of ATP synthesis. ATP synthesis and hydrolysis, as well as the Pi–HOH and ATP–HOH exchanges, are all inhibited by oligomycin [82,83], and hence are believed to be closely related at the level of reaction mechanism. The oligomycin sensitivity suggests that these reactions occur at the same or related enzyme catalytic sites, and hence the mechanisms of the partial reactions of the oxygen exchanges will help us to gain a better understanding of the overall chemical reaction mechanism of ATP synthesis/hydrolysis. Any mechanism of ATP synthesis/hydrolysis must account for the faster Pi–HOH and ATP–HOH oxygen exchanges.

#### 2.2.1. Derivation of Limitations on the Rates of Oxygen Exchange

Certain limitations on the rates of Pi–HOH and ATP–HOH oxygen exchanges relative to the Pi–ATP exchange can be derived for a single point/route of water entry/exchange based on consideration of the two limiting cases, as follows:

(i). Release of Pi is limiting. In this case, bound ATP can rapidly exchange with ^18^O in H^18^OH, which will cause the label to appear in ATP or subsequently in P_i_. However, each inorganic phosphate molecule released can contain at most four oxygens from ^18^O labeled water. Thus, in this case, the rate of ATP–HOH exchange can be high relative to the Pi–ATP exchange, but the rate of Pi–HOH exchange relative to the Pi–ATP exchange cannot be greater than four;
(ii). Release of ATP is limiting. In such a case, tightly bound P_i_ can rapidly exchange with the oxygens of ^18^O water, but the γ-phosphate of ATP can contain at most three labeled oxygens. Thus, in this situation, the rate of Pi–HOH exchange can be high relative to the Pi–ATP exchange, but the ratio of the rate of ATP–HOH exchange to that of the Pi–ATP exchange cannot exceed three.

#### 2.2.2. Measurement of the Relative Rates of Isotope Exchanges and Difficulties of Their Rationalization by the Existing Model

The initial experiments from Boyer’s group yielded a rate of the Pi–HOH exchange approximately 20-fold greater compared to the Pi–ATP exchange rate and the net rate of ATP synthesis in mitochondria [84]. Subsequently, these were confirmed by comprehensive experimentation from Lehninger’s group, who found the Pi–HOH rates to be between 10 and 25 times faster than the rate of the Pi–ATP isotope exchange, depending on the conditions employed [85]. Further new kinetic information emerged from this study. Lehninger and colleagues [85] also measured the ATP–HOH oxygen exchange rates and found these rates to be 5–15 times more rapid relative to the rates of the Pi–ATP exchange, again depending on the conditions used in the experiments. The unexpected finding now was that the rates of both the Pi–HOH and ATP–HOH exchanges are higher than the theoretical limits derived above (Section 2.2.1) relative to the rate of the Pi–ATP exchange.

Following the measurement of the relative rates of the isotope exchanges noted above, these experiments were repeated during the next decade. Boyer reported a ratio of 12:6:1 for the Pi–HOH:ATP–HOH:Pi–ATP exchange rates in mitochondria [59]. Our measured values of the relative rates of the Pi–HOH:ATP–HOH:Pi–ATP exchanges in submitochondrial particles are 18:6:1 (Figure 2), and from the short-time experiments, as high as 48:16:1, respectively, at high ATP:ADP concentrations. Hence the kinetic feature of relatively high rates of both the Pi–HOH and ATP–HOH oxygen exchanges (greater by a factor of ten) with respect to the Pi–ATP isotope exchanges was confirmed. This led to acute difficulties in reconciling the confirmed experimental observations vis-à-vis the model/scheme employed in rationalizing the general characteristics of the oxygen exchanges [59].

A major limitation of Boyer’s model of oxygen exchange was that it involved only a single site/mode of entry of water oxygen [59]. Hence, relative to the Pi–ATP exchange, it could readily explain one oxygen exchange reaction, say the Pi–HOH exchange, taking place at a rate well above the limiting value of four derived in Section 2.2.1 with respect to the Pi–ATP exchange rate. This is because it could be postulated that bound Pi can continue to exchange with the HOH through the single entry point, and that the release of labeled Pi can occur frequently (compared to ATP) from the catalytic site, and hence such a medium Pi–HOH exchange could cause the relative rates to exceed the 4:1 ratio derived above. However, from the theoretical analysis, we can infer that it is impossible for the rates of both the oxygen exchange reactions to be high simultaneously, relative to the rate of the Pi–ATP exchange/ATP synthesis. Looking at it mechanistically, it is impossible for a single point of water entry to raise the rates of two oxygen exchanges at the same time, i.e., of water with both Pi and ATP bound to catalytic sites (relative to the rate of the Pi–ATP exchange/ATP synthesis) well above the theoretical limits derived in Section 2.2.1. Hence, we conclude that the high rates of both the Pi–HOH and the ATP–HOH exchanges relative to the Pi–ATP exchange found in the experiments cannot be rationalized by Boyer’s scheme in the binding change mechanism [59].

#### 2.2.3. Possible Explanation of the Kinetic Data on Relative Rates of Oxygen Exchanges

The ultra-high rate of the Pi–HOH exchange and the high rate of the ATP–HOH exchange compared to the Pi–ATP exchange, as discussed in Section 2.2.2, argue strongly against the phenomenological scheme postulated by the binding change mechanism [59] as the explanation for the exchange reactions. Possible alternative explanations are that either a mechanism involving a single site or route of entry of water oxygen does not apply to oxidative phosphorylation, or that much of the Pi–HOH exchange occurs via a reaction separate from the postulated reversible phosphorylation reaction of oxidative phosphorylation, or both, since the alternatives are not mutually exclusive. In fact, in addition to the results shown in Figure 2 and discussed in Section 2.1, and the evidence cited in Section 2.2.2, there is other biochemical evidence that supports the separate character of much of the Pi–HOH exchange. For instance, at low concentration levels of 0.2 μg/mg protein, oligomycin is known to stimulate oxidative phosphorylation as well as the Pi–ATP exchange in particles [83]; however, at this low dosage, it inhibits the Pi–HOH exchange [82]. This simultaneous inhibition of the Pi–HOH exchange and the stimulation of the Pi–ATP exchange and phosphorylation offer further strong support to the proposal that much of the Pi–HOH exchange occurs by a reaction separate from the hypothesized reversal of ATP synthesis at the catalytic site.

It should also be noted that the meeting of the nucleotide requirement of the Pi–HOH exchange by ATP provides no support to the phenomenological oxygen exchange scheme of the binding change mechanism [59] requiring reversal of the phosphorylation reaction of oxidative phosphorylation. Rather, it favors the proposal that a separate means (e.g., ATP hydrolysis) of driving the intermediate Pi–HOH exchange is present in mitochondrial systems—as proposed in this work—and that the activated or energized intermediate state, X, can also be formed essentially irreversibly by ATP cleavage, giving rise to an intermediate Pi–HOH exchange without a concomitant Pi–ATP or intermediate ATP–HOH exchange.

#### 2.2.4. Resolution of the Apparently Conflicting Experimental Data and Discussion of Other Key Mechanistic Aspects of ATP Synthesis/Hydrolysis

The studies outlined in Section 2.2.3 on the Pi–HOH oxygen exchange reactions in reality serve to help distinguish between pathways for ATP synthesis and ATP hydrolysis/utilization. Boyer and colleagues interpreted the exchange observed in their system as a medium Pi–HOH exchange. However, if the residual Pi–HOH exchange in the experiments on ATP hydrolysis in the presence of an ATP regenerating system was actually an intermediate Pi–HOH exchange rather than a medium Pi–HOH exchange, then the apparently conflicting data can be readily reconciled. This interpretation would also be consistent with subsequent experimental work that shows the Pi–HOH exchange in the presence of an ATP regenerating system to be free from arsenate inhibition [57,86,87]. In summary then, there are two types of mitochondrial Pi–HOH exchanges, (i) an intermediate Pi–HOH exchange that requires no medium Pi and in which the energized state is created by an essentially irreversible ATP hydrolysis [30,31], and (ii) a medium Pi–HOH exchange that requires ADP and medium Pi, and involves the same or a similar activated or energized state X, but one that arises now from conformational changes in F_1_ due to ion translocation processes in F_O_ and the resulting rotation of the γ-subunit [31,33].

The differential effects of the very potent nitrosalicylanilide uncoupler of oxidative phosphorylation on the oxygen exchanges, S-13, and especially the unusual resistance of the Pi–HOH exchange to it are particularly well documented in the literature on the subject [59]. The classical observations of Drysdale and Cohn [88] on the milder uncoupler 2,4-dinitrophenol show that concentrations well in excess of those needed to uncouple oxidative phosphorylation by intact mitochondria are needed to inhibit the Pi–HOH exchange. We have previously studied coupling [89] and uncoupling [46,90] activities in ATP synthesis. Figure 3 characterizes the 2,4-dinitrophenol dependence of the various exchanges catalyzed by submitochondrial particles. Here, 0.2 mM 2,4-dinitrophenol almost completely eliminated the Pi–ATP and ATP–HOH exchanges, while an appreciable (intermediate) Pi–HOH exchange continued to occur at this concentration. At a 1 mM uncoupler concentration, >25% of the Pi–HOH exchange activity remained (Figure 3), and a twenty-fold higher concentration of 2,4-dinitrophenol was required to inhibit 90% of the intermediate Pi–HOH exchange compared to the concentration required for complete disappearance of the Pi–ATP exchange. This important feature of the continued presence of a large, prominent uncoupler-resistant intermediate Pi–HOH oxygen exchange reaction accompanying ATP hydrolysis is incompatible with the reversible scheme for oxygen exchange ADP + Pi + H^+^ + X ↔ ATP + HOH, as well as with the explanations provided for its occurrence [88].

The intermediate Pi–HOH exchange was shown to display unusual insensitivity to uncouplers of oxidative phosphorylation; for example, take the cases of S-13 [59] and 2,4-dinitrophenol (Figure 3). Thus, the puzzling aspect was that although the uncoupler allowed oxidation to proceed without net ATP synthesis, the rapid exchange of phosphate and water oxygen atoms continued. Boyer felt that these results could be explained if the energy of the ion gradients was used not to synthesize the ATP molecule, but instead was used to cause the release of a tightly bound ATP. The reversible formation of the tightly bound ATP molecule could continue at the catalytic site without involving the energy of the ion gradients, and give rise to the uncoupler-insensitive oxygen exchange [59,60]. However, this conclusion of the binding change mechanism was false and misleading.

The above explanation of the binding change mechanism was not the only possible explanation for the continued occurrence of an uncoupler-insensitive intermediate Pi–HOH exchange. Since the intermediate Pi–HOH exchange can be catalyzed in mitochondria and particles by a separate reaction (e.g., irreversible ATP cleavage) independent from the formation of ATP by the reactions of oxidative phosphorylation, the intermediate Pi–HOH exchange would indeed display the relative insensitivity to uncouplers (Figure 3) compared to the Pi–ATP and ATP–HOH exchanges characteristic of oxidative phosphorylation. We would thus be hopelessly misled if we did not consider the separate character of the intermediate Pi–HOH exchange compared to the others, i.e., the Pi–ATP and ATP–HOH exchanges, and continued to believe that the intermediate Pi–HOH exchange occurred due to the reversible phosphorylation reaction.

Boyer ignored the separateness of much of the Pi–HOH exchange in the development of the binding change mechanism, and postulated the involvement of ADP as a substrate in a reversible ATP formation reaction from ADP + Pi as the mechanistic basis for the apparent absolute requirement of ADP for the (medium) Pi–HOH exchange [60]. However, a requirement of ADP for the medium Pi–HOH exchange could also be indicating the possibility of a pentacovalent intermediate as a participant in the reaction involving water formation in oxidative phosphorylation. In other words, ADP permits the formation of a pentacoordinated phosphorus derivative that exchanges oxygen with water. This hypothesis would rationalize the apparent absolute requirement for ADP in the medium Pi–HOH exchange; further, such a proposal would also be consistent with an intermediate Pi–HOH exchange separate from the “reversible” phosphorylation reaction. The Pi–ATP and intermediate ATP–HOH exchanges are then seen as being diagnostic of ATP synthesis and oxidative phosphorylation (with the intermediate ATP–HOH exchange being unique to oxidative phosphorylation and photophosphorylation), while the intermediate Pi–HOH exchange can be concluded to be catalyzed solely by ATP hydrolysis. However, the same energized ADP.P_i_ intermediate state in which medium and intermediate Pi–HOH exchanges occur (e.g., in the L-site in either mode [31,33,77,78]) can be generated separately by the different driving forces in each mode, i.e., by ion translocation in ATP synthesis [11,15,24,25,26,31,33,36,46,67,68,69,71,73] and by ATP itself in the process of ATP hydrolysis [30,31,77,78].

**Figure 4:**
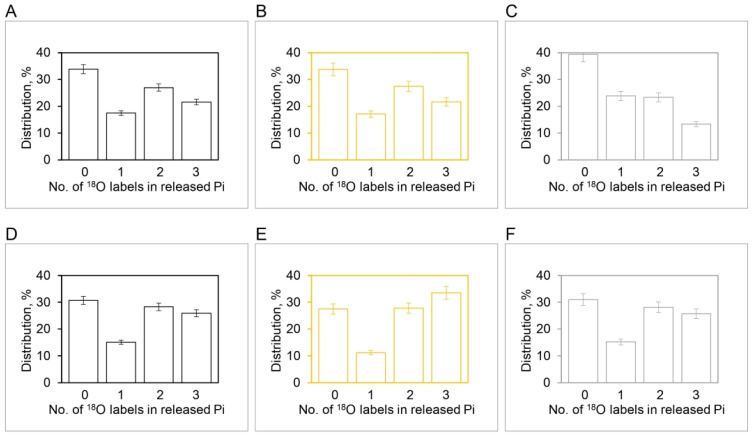
Experimental and theoretical [^18^O]Pi distributions during ATP hydrolysis by mitochondrial F_1_ at 3 µM ATP (average ^18^O/P = 2.10: **A**–**C**) and 5 µM ATP (average ^18^O/P = 1.85: **D**–**F**) based on experiments in the region of intermediate ATP concentrations (mean ± SD, *n* = 6). Experimental distributions at 3 µM ATP (**A**), theoretical distributions at 3 µM ATP with n=2 (**B**), and theoretical distributions at 3 µM ATP with n=3 (**C**). Experimental distributions at 5 µM ATP (**D**), theoretical distributions at 5 µM ATP with n=2 (**E**), and theoretical distributions at 5 µM ATP with n=3 (**F**). The initial distribution of the ^18^O label in the γ-phosphoryl group of ATP in our experiments measured as follows—3 ^18^O: 57%; 2 ^18^O: 17%; 1 ^18^O: 2%; 0 ^18^O: 24%.

Figure 4 shows the results of our experiments on hydrolysis by mitochondrial F_1_ on the [^18^O]Pi distributions at intermediate ATP concentrations. The results show that between 5 µM and 3 µM ATP, the number of catalytic sites, n, mediating oxygen exchange at the same time switches from three (Figure 4D–F) to two (Figure 4A–C), respectively. Hence there is a transition from n=3 to n=2 between 3 and 5 µM ATP, and only then can the [^18^O]Pi distributions shown in Figure 4 be predicted exactly [91].

The presence of multiple sites of water entry in ATP hydrolysis depending on ATP (n=3 for ATP > 3–5 µM, and n=2 for ATP < 3–5 µM) (Figure 4) readily explains the observations of the high rates of both the Pi–HOH exchange and the ATP–HOH exchange (with respect to the rate of the Pi–ATP exchange) discussed at length in Section 2.2.2, as also the fact that these rates were found to be above the theoretical limits derived in Section 2.2.1 in a number of carefully carried out experiments. The rapid intermediate Pi–HOH and ATP–HOH exchanges are also explained if the exchanges do not involve the overall dynamic reversal of, but instead occur because of the lack of, absolute spatial selectivity in the binding of oxygen atoms of tetrahedral phosphate to enzyme [57,79,91].

The principal similarities and differences between the Boyerean and Nathean mechanisms of oxygen exchange may be summarized—in their overall features, with details eschewed, it ought to be stressed—by considering the scheme for ATP hydrolysis at an F_1_ catalytic site, as shown by Equation (2):

(2) $$HOH+E.ATP\;\begin{array}{c} k_{h}\\ ⇄\\ k_{r} \end{array}E.ADP.Pi\;\overset{k_{t}}{\to }\;E.ADP$$

where $k_{h}$ governs the rate constant of hydrolysis, $k_{r}$ refers to the rate constant of the reverse cleavage step, and $k_{t}$ represents the irreversible product release step that terminates the intermediate Pi–HOH exchange. One oxygen atom from water appears in each molecule of Pi released by the hydrolysis reaction in both models, as shown by Equation (1). Both models require a spatial rearrangement of the oxygen ligands of phosphorus either in a single catalytic site (Boyer, with n=1) or at >1 catalytic sites (Nath, with n=2 or n=3 depending on [ATP]). This is due to the fact that for exchange to occur, the oxygen atom that is lost by the tightly bound E.ATP or tightly bound E.ADP.Pi intermediates during exchange that gets incorporated into the Pi released upon termination of the exchange has to be different from the oxygen atom gained from water. This can happen, for example, by rotation or torsion of the bound Pi in the E.ADP.Pi state or of the γ-phosphoryl group of ATP in the E.ATP state in the active site(s). It is also required by both models that the rate of rotation and/or permutation of the oxygens around the phosphorus atom is sufficiently fast so that it does not limit the rate of oxygen exchange, i.e., there is no restriction to ligand rotation/permutation in the active site(s). In both models, water has unrestricted access to the catalytic site or sites, and all oxygens on the bound Pi/ATP are considered to be chemically equivalent. However, the explanations for the occurrence of the intermediate Pi–HOH and ATP–HOH exchanges are different between the two models.

In Boyer’s model, the reverse cleavage step governed by the rate constant $k_{r}$ is considered to be relatively fast compared with the rate-limiting product release step with rate constant $k_{t}$. Hence several cycles of cleavage and reverse cleavage (Equation (2)) are envisaged to occur before a molecule of Pi is released into the medium, and such reversals are considered to be responsible for the observed oxygen exchange. The extent of oxygen exchange increases with the number of reversals, R, of bound hydrolysis prior to Pi release, with each reversal having a 3/4 probability of exchange. R is given by the equation

(3) $$R=\frac{k_{r}}{k_{t}}$$

and is related to the partition coefficient, Pc=kr/kr+kt=41−1/O18/Pi/3, by the relationship

(4) $$R=\frac{P_{c}}{1-P_{c}}$$

In Nath’s torsional mechanism, the rate constant of ligand permutation/exchange, $k_{e}$, per oxygen atom per second at a single catalytic site is considered to be responsible for the intermediate Pi–HOH and ATP–HOH isotope exchanges. $k_{e}$ is relatively large compared with the rate-limiting product release step with rate constant $k_{t}$. The greater the lifetime of the exchanging intermediates, i.e., the time t available for permutation and exchange of ligands in the tightly bound E.ATP and E.ADP.Pi states in the catalytic sites, for example t~1/[ATP] or $t\sim 1/k_{t}$, the greater is the extent of oxygen exchange. There is no need for repeated cycles of cleavage and reverse cleavage of ATP to explain the origin of the oxygen exchanges in Nath’s mechanism, unlike in Boyer’s model. It is therefore a true exchange that does not involve the overall reversal of catalysis. Since ATP is not cleaved and re-cleaved, i.e., the relevant P–O bond is not broken and re-formed repeatedly, the Pi–HOH and ATP–HOH oxygen exchanges occur at a significantly faster rate compared to the rate of the Pi–ATP exchange that requires complete reversal of the catalysis mechanism. Further, the exchanges can continuously occur at multiple catalytic sites that contain either bound ATP or bound ADP.Pi, a factor that also contributes to their rapid nature.

A general kinetic approach to oxygen exchange—that makes no specific assumptions about mechanism—has been developed for ATP synthesis [57], and most recently for the intermediate Pi–HOH exchange accompanying ATP hydrolysis [91]. A principal result of the latter treatment (Equation (6) in Ref. [91]) is reproduced here as Equation (5)

(5) ln0.751−O18/Pi4=kt

in which k is the average apparent rate of oxygen exchange per s of turnover time, t, per catalytic site. The expression for the amount of oxygen exchange, kt, is given by Equation (5) irrespective of whether the exchange occurred prior to the cleavage of bound ATP, at an intermediate stage of hydrolysis, or following hydrolysis due to exchange between the oxygen of tightly bound Pi and that of medium water [91]. Equation (5) also satisfactorily accounts for the initial conditions, and mathematically corrects for any ^18^O of water already incorporated into a phosphate group [91].

A plot of ln1/(1−O18/Pi4) versus t yields a slope k for the prominent intermediate Pi–HOH exchange mediated by F_1_-ATPase of 10.5±0.1 s^−1^ site^−1^ [91], which is a measure of the average number of exchanges per s of turnover time per catalytic site. Such a plot leads to an intercept on the ordinate of 0.75 instead of 1.0 (see Equation (5)). This is because one ^18^O atom exchanges very fast and is incorporated into Pi by the hydrolytic cleavage reaction (Equation (1)) almost at t=0. Each of the other three oxygens exchanged with the same rate constant $k_{e}=k/3=3.5\pm 0.033$ s^−1^ O^−1^ site^−1^. Note that the amount of oxygen exchange kt in Nath’s model of oxygen exchange (Equation (5)) is a continuous function of ^18^O incorporation and exchange into a Pi molecule.

A different equation—the analog of Equation (5) for oxygen exchange according to Nath’s torsional mechanism of ATP synthesis/hydrolysis—for the amount of water oxygens incorporated into a Pi molecule can be written for Boyer’s model. It assumes that cleavage and re-cleavage cycles are responsible for the exchange based on the number of times, N that the cleavage step has occurred, with a 3/4 factor of probability for loss/gain of the ^18^O label with each reversal. An expression for the number of water oxygens in a Pi molecule can be written as (Equation (6)):

(6) O18Pi =∑N=1N3/4N−1

It ought to be emphasized that the experimental record of ^18^O/Pi obtained by mass spectroscopic methods as a function of medium ATP concentration produces a continuous variation of the number of water oxygens incorporated into a Pi molecule (Figure 2 in Ref. [91]). However, Equation (6) written for Boyer’s mechanism models the oxygen exchange in discrete steps. An approximate relationship between the two estimates for the oxygen exchange is given by

(7) $$1+3k_{e}t=N$$

Hence, a major difference between the two models of oxygen exchange can be summarized as follows. In Boyer’s model, the origin of oxygen exchange arises from the hypothesis of the occurrence of multiple forward and reverse cycles for the ATP cleavage reaction. By contrast, in Nath’s model, oxygen exchange occurs due to ligand permutation in the MgATP/MgADP.Pi-filled catalytic sites of the F_O_F_1_/F_1_-ATPase enzyme without the requirement of ATP bond breaking and bond re-forming events.

It should be clear from the aforementioned discussion that the number of reversals, R, of bound ATP hydrolysis prior to Pi release takes on the role of a fitted parameter that models oxygen exchange. There is no independent verification that the reaction actually reverses itself R times. In other words, although the procedure of Equation (4) yields an evaluation of R, assuming that Boyer’s model of reaction reversal to explain oxygen exchange (Equation (2)) is valid, it provides no means of establishing whether Equation (2), involving reversal, is, in fact, a valid description of the oxygen exchange process.

The above statement can be quantified. In terms of Equation (6), N is large (>15) to model oxygen exchange at micromolar ATP. At the lowest concentration of ATP (0.1 µM) used in our oxygen exchange studies [91], the value of R would have to be >50 to explain our ^18^O/Pi distributions measured by Boyer’s model. At lower ATP (~0.03 µM), the reaction would need to involve even more reversals, R> 400, as already mentioned. By contrast, in Nath’s approach, the extent of oxygen exchange, kt, is directly obtained from the experimental measurements of the overall O18/Pi (Equation (5)). The distributions of all four Pi isotopomers containing 0, 1, 2, and 3 ^18^O labels are modeled solely in terms of partition functions containing *kt* [91]. The complete distributions of the 4 Pi species have been shown to be accurately predicted by Nath’s model of oxygen exchange throughout the entire course of the exchange reactions over a 50,000-fold ATP concentration range (0.1 µM–5 mM), without employing fitted parameters [91]. We consider this as a critical test for any proposed model of oxygen exchange.

More important, what are the characteristics of oxygen exchange at physiological ATP concentrations (~1  mM)? In fact, already at the upper limit (5 µM) of the intermediate ATP regime explored in Figure 4, $P_{c}<0.5$, and regarding the number of spontaneous reversals, R<1. For the physiological ATP concentration range between 1 mM and 5 mM [91], both $P_{c}$ and R work out to be as small as ~0.02, i.e., the number of postulated reaction reversals, R≪1, for Boyer’s model of oxygen exchange. In other words, the ATP hydrolysis catalyzed by F_O_F_1_/F_1_-ATPase is essentially irreversible at physiological conditions, exactly as first postulated by Nath’s torsional mechanism of ATP synthesis/hydrolysis [33], and subsequently quantitated for both ATP synthesis [57] and ATP hydrolysis modes of functioning [77,91]. Hence it is not useful to characterize the oxygen exchange occurring at physiological substrate concentrations using Boyer’s model.

From the standpoint of this work:

(i). The meeting of the nucleotide requirement for the intermediate Pi–HOH exchange by ATP, but not by ADP, with the latter postulated as an obligatory requirement by the binding change mechanism [81], goes against the mechanism (Section 2.1);
(ii). The concomitant enhancement (Section 2.2.2) above theoretical limits of both the intermediate Pi–HOH and ATP–HOH exchanges with respect to the Pi–ATP exchange (Section 2.2.1) is impossible to explain using Boyer’s simple model containing only a single route of water entry.

However, there are other major difficulties for the binding change mechanism, which are pointed out below:

(iii). The equations of the binding change mechanism have not been cast in a model-independent way [57,91]. There is no independent verification of the R>400 reversals postulated to occur as a result of the mechanism at low substrate concentrations during ATP synthesis/hydrolysis. At physiological ATP, R≪1 for the F_1_-ATPase, leading in essence to an irreversible cleavage of ATP. As formulated, R has to be an integer, and it is difficult to interpret a non-integer value of R in molecular terms;
(iv). For ATP hydrolysis by the F_O_F_1_-ATPase, the incorporation of water oxygens into ATP/Pi that arises from the postulated repeated cycles of reversal of the ATP formation reaction has to also be accompanied by a reversal in the direction of H^+^ translocation across the membrane. However, such a rapid fluctuation/change in direction of the electrochemical ion gradients, and such cycles of bidirectional ion movement, have never been demonstrated in any biochemical system. It was stated by Boyer in 1997 that this “remains an important question” [92], but it has not been addressed, let alone answered, even after the passage of >25 years;
(v). Point (iv) is further exacerbated by the fact that mechanical aspects of the ATP synthase motor also need to be included in the analysis. We stated that, “it is imperative to relate the chemical kinetics (the arrows representing an elementary step in the kinetic scheme) to the mechanical aspects (structure and dynamics of the molecular machine). It is very difficult to conceive how a unidirectional, discrete motion can take place by a reversible mode of catalysis (E.ATP ⇄ E.ADP.P_i_), i.e., how can a subunit of a single enzyme molecule oscillate back and forth in the presence of a driving force in one direction? This irreversibility of operation in a single molecule mode contradicts the fundamental tenet of the binding change mechanism that ATP synthesis occurs reversibly (and spontaneously) in a catalytic site of the enzyme” [33];
(vi). During the operation of a molecular motor at physiological conditions, a species is encouraged to bind to its site on the enzyme mainly to undergo the reaction to the product, and thereafter immediately unbind and release the product. There is no logical explanation as to why it should bind and be released before its conversion to product, or why a reaction should move back and forth numerous times before its product is finally released. Nor can this be considered an efficient mode of operation of the system;
(vii). The binding change mechanism proposed a catalytic site at which $K_{eq}\sim 1$ during steady-state catalysis by the ATP synthase/F_1_-ATPase [59]. Later, in one of his last papers on the subject, Boyer acknowledged that “during hydrolysis the quasi-equilibrium may be shifted toward bound ADP so that essentially only ADP will be released” [61]. It further added that “how this could be achieved is not known” [61]. We are none the wiser twenty years on after that statement was made;
(viii). The bisite binding change mechanism revealed fundamental flaws, in that low-affinity F_1_ catalytic sites were occupied by bound MgATP, while higher-affinity catalytic sites were left unfilled during the catalysis of ATP hydrolysis [33];
(ix). While “unisite” catalysis and its acceleration by chase ATP binding at a second catalytic site has been amply demonstrated [78], there has been no report of “unisite” ATP synthesis. Nor has the acceleration of the rate of ATP synthesis been demonstrated to achieve the physiological catalysis of synthesis. In fact, classical biochemical experiments on the mitochondrial F_1_ using radioactive probes showed that rapid steady-state ATP synthesis is achieved by the enzyme only after the filling of three catalytic sites [93];
(x). Pioneering biochemical studies by Senior and coworkers [94] using fluorescent probes have conclusively demonstrated that trisite catalysis is the true operating mode of steady-state V_max_ hydrolysis by F_1_-ATPase. The technologically sophisticated single-molecule studies of Kinosita and coworkers on F_1_-ATPase offer further support to a trisite mode of ATP hydrolysis from nanomolar to millimolar ATP concentrations [95]. Finally, high-resolution X-ray structures of F_1_-ATPase that visualized the transition state have proven beyond doubt that Mg-nucleotide binds to all three β-catalytic sites of the F_1_-ATPase during catalysis [76]. It is very difficult, if not impossible, to explain these important findings based on bisite models of catalysis, such as the binding change mechanism;
(xi). It was very important to distinguish between bisite activation and trisite catalysis during the process of ATP hydrolysis. The binding change mechanism did not do that, which, in retrospect, can be considered a major shortcoming. Trisite catalysis with bisite activation in ATP hydrolysis by F_1_-ATPase has been explained only very recently [77,91];
(xii). The cleavage of bonds followed by their re-formation by irregular processes leads to intermediates of lower coordination number. Hence, such processes do not satisfy the general chemical concept of the preservation of coordination number. The latter is only possible if the molecular skeleton is flexible, i.e., the numbered positions on the skeleton (such as the trigonal bipyramid skeleton) can be interconverted with the maintenance of coordination numbers via the deformation of bond angles or rotation about bonds (dynamic skeletal symmetry). Hence there is no chemical necessity to propose mechanisms involving bond breaking and bond re-forming processes as done by the binding change mechanism in order to rationalize the oxygen exchange reactions. We shall further discuss this concept, which is of great importance to phosphorus chemistry, in Section 2.2.5 and Section 2.2.6.

The above dozen aspects show the multiple defects and fundamental weaknesses in the theoretical framework of the binding change mechanism. They also reveal the serious inconsistencies/contradictions in a vast amount of the experimental data, as reviewed previously, and shown to be overcome by the torsional mechanism [30,31,32,33,34,77,78,91].

The concepts developed here for oxygen exchange catalyzed by the ATPases have a wider, and possibly evolutionary, biological significance. For instance, the inorganic pyrophosphatases have been shown previously to catalyze similar oxygen exchange processes [96,97]. Pyrophosphate (PPi) probably served as the main energy carrier in the “PPi world” under the chemical conditions that prevailed in the early history of life on Earth, and preceded the “ATP world”. Relics of this ancient membrane-based energy transduction that use PPi in lieu of ATP are found in bacteria, archaea, and plants [96,97,98,99,100]. The medium and intermediate Pi–HOH/ATP–HOH exchange catalyzed by inorganic phosphatase, analogous to the exchanges by the F_1_-ATPase/F_O_F_1_-ATP synthase [57,91] at physiological conditions, are characterized by values of $P_{c}<0.5$ and R<1 [96]. Hence the aforementioned concepts for analysis of the exchange reactions are applicable to the inorganic pyrophosphatases also.

The X-ray crystal structures of a H^+^-translocating pyrophosphatase [98] and a Na^+^-translocating pyrophosphatase [99] have offered important structural insights. Yet they have not solved the mechanistic problems [100], a situation reminiscent of, and similar to, that prevailing in the field of ATP synthesis/hydrolysis [78]. The mechanism of Lin et al. [99] suggests that H^+^ translocation succeeds or occurs simultaneously with the hydrolysis of PPi. Distinction between these two possibilities was not possible [99]. On the other hand, the alternative mechanism of Kellosalo et al. [100] proposes that the H^+^ ion is transported across the membrane due to PPi binding energy, and that the hydrolysis is required only to complete the catalytic cycle so that a new transport cycle can begin, a scenario similar to that proposed within Boyer’s mechanism [59,60].

Nath’s molecular theory of biological energy transduction and ATP synthesis/hydrolysis [30,31,57,77,91] helps resolve these recalcitrant mechanistic problems both in the “ATP world” and in the “PPi world”. The theory definitively rules out the possibility that the ion transport is associated with the ATP/PPi binding step in a single-molecule turnover mode. Nor is it sufficient to simply hydrolyze the ATP/PPi in a cleavage step at the catalytic site and expect ion translocation across the membrane to occur. The theory clearly stipulates that after the elementary chemical act of the hydrolytic cleavage step, it is essential to unbind the Pi and move it away, and finally eject it out and release it into the surrounding medium, a process that is associated with, and donates energy for, the transmembrane cation transport.

As to the role of anions in the inhibition/stimulation of the ATPases, a vast, complex, and often contradictory section of the literature has accumulated on the subject since the pioneering classical work of Ebel and Lardy [101], all of which cannot be covered here. We have studied the effects of various anions on ATP synthesis/hydrolysis in animal mitochondria and plant chloroplasts (for a review, see [102]). Sulfate does not have a significant effect on the stimulation/inhibition of ATP hydrolysis in the presence of high Pi, especially the 10 mM used in our experiments. There was no inhibition of the initial rates of hydrolysis by 5 mM fluoride in our preparations. We have recently published a large work that contains within it a detailed description of the molecular mechanism of azide inhibition of ATP hydrolysis by F_1_-ATPase, a subject of great pharmaceutical importance [77]. In the present work, we were primarily concerned with employing the same buffer and medium conditions—i.e., 250 mM sucrose and 50 mM Tris sulfate, pH 7.5—as used in the studies on submitochondrial particles of Jones and Boyer [81] which had previously postulated an obligatory requirement of ADP for the occurrence of the Pi–HOH exchange in oxidative phosphorylation. We have also ensured that all the exchange reactions are compared under identical conditions on the same reaction mixture.

A number of other controls were run. For example, in the absence of added ADP, the submitochondrial particles revealed a small but detectable apparent Pi–HOH exchange. At 5 mM Mg^2+^ in the presence of 10 mM glucose and hexokinase (0.1 mg mL^−1^), but without added ADP, the exchange measured <2.0% of the total, and incorporated <0.5 µatoms oxygen out of 25 µatoms oxygen exchanged at 1 mM ADP in the absence of hexokinase. This negligible ADP-independent ^18^O incorporation into Pi was oligomycin-insensitive. It was not obliterated by the addition of oligomycin at a concentration of 1 µg mL^−1^. The hydrolysis of minute amounts of other phosphate compounds present in the submitochondrial preparations could be responsible for this small apparent exchange that is caused by reactions unrelated to oxidative phosphorylation. The addition of imidodiphosphate (10 µM) had no effect on the intermediate Pi–HOH exchange. The relative insensitivity of the intermediate Pi–HOH exchange to 2,4-dinitrophenol and the high phenol concentration required to abolish the exchange found in this work on particles (Figure 3) is also demonstrable in intact mitochondria. Above all, similar results on the continuation of a robust Pi–HOH exchange with mitochondria and its relative lack of inhibition have been observed with several uncouplers. These include carbonyl cyanide 3-chlorophenylhydrazone (CCCP), and the very potent uncoupler 5-chloro-3-t-butyl-2′-chloro-4′-nitrosalicylanilide (S–13) [59], in addition to 2,4-dintrophenol. These results support the conclusion that exchange in particles results from the intrinsic activity of well-coupled mitochondria.

In conclusion, Nath’s molecular theory of biological energy transduction and ATP synthesis/hydrolysis has multifarious applications to several biosystems, such as the ATP synthase/rotary ATPases, muscle myosins, unconventional myosins and kinesins, inorganic pyrophosphatases, DNA helicases, RNA polymerases, etc. It is a general and unified molecular-level theory. The complete cycle of energy transduction can be understood at the molecular level, and the mechanistic problems resolved with finality, if we have the will to take recourse to the theory [30,31,57,77,91].

#### 2.2.5. New Concepts for Rationalization and Explanation of Oxygen Exchange in Oxidative Phosphorylation and Photophosphorylation

**Figure 5:**
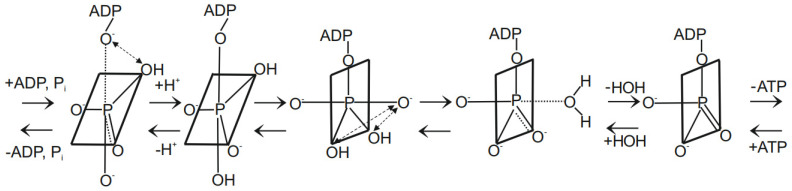
Reaction scheme at the β-catalytic site of the F_1_ portion of ATP synthase. The scheme illustrates the essential intermediate reaction stages in ATP synthesis (forward arrow) and hydrolysis (reverse arrow) and explains the occurrence of the Pi–HOH and ATP–HOH oxygen exchanges (see the arrowed dashed lines) by permutation of ligands about the phosphorus center of the trigonal bipyramidal pentacovalent reaction intermediates. Note also the interchange between apical and equatorial positions of the bulky ADP moiety during the transformation. Mg^2+^ (not shown) with its coordination number of six is also expected to play a crucial role in catalysis; after formation and binding of the bidentate β,γ Mg.ADP.P_i_/MgATP in the catalytic site, a four-fold coordination and interaction with amino acid side chains and water molecules can assist the rearrangement of ligands shown here. Further details are given in Section 2.2.5 of the text.

Given the above characteristic of the catalytic site in F_1_, it is then clear that ligand permutation can readily take place in an intermediate state, and the oxygen ligands can interchange their positions about the phosphorus center. We have proposed here and previously [57,79] that such a permutation of ligands is the fundamental cause for the occurrence of the Pi–HOH and ATP–HOH oxygen exchanges (as opposed to multiple spontaneous reversals of ATP synthesis in the catalytic site). Further, we envisage that such ligand permutation occurs under conditions of tight electrostatic interactions with catalytic site groups, such that the electrostatic free energy required (for the process of ATP synthesis) or released (during the process of ATP hydrolysis) can be readily transferred from/to the enzyme, e.g., from/into the torsional energy stored in the γ-subunit of ATP synthase, as per the postulates of the torsional mechanism of energy transduction and ATP synthesis/hydrolysis, and the unified theory of ATP synthesis/hydrolysis [31,33,63,64]. Mg^2+^ would be expected to play a critical role in this process via coordination with the negatively charged oxygen ligands of P_i_/ADP/ATP, as suggested previously [63,64,103,104,105,106]. A mechanistic scheme incorporating the above concepts that depicts the essential intermediate stages of the ATP synthesis/hydrolysis reaction and shows the occurrence of the oxygen exchanges by permutation of the ligands of phosphorus is given in Figure 5.

The transformations and the exchange processes in the scheme (Figure 5) rely on simple regular processes involving permutational rearrangement by deformation operations on the flexible pentacoordinated phosphorus skeleton, instead of invoking bond-breaking irregular processes as in the binding change mechanism. In our view, the simpler options must be exhausted before resorting to the proposal of the more drastic latter mechanisms. In fact, intramolecular ligand rotation/twist and ligand permutation is an intrinsic property of the flexible γ-phosphorus skeleton.

**Figure 6:**
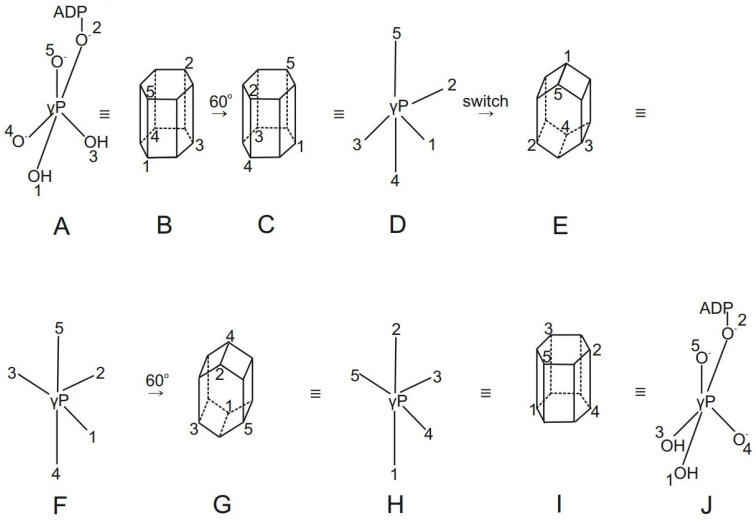
Illustration of the proposed reaction mechanism during energy-dependent conformational transitions at the β-catalytic sites in the F_1_ portion of ATP synthase. The mechanism incorporates 120° relative ligand rotation/twist and ligand permutation, and proceeds with inversion stereochemistry at the γ-phosphorus atom, in accordance with experimental results [110,111]. A hexagonal prism representation is also included to assist the clear visualization and to clarify the stereochemical situation. Simple rules to obtain the correct conformation can be readily formulated. Thus, the pair ligands (one axial and one equatorial ligand, e.g., ligands 2 and 5 in (**A**), and ligands 1 and 4 in panel (**F**)) simply exchange positions. In the case of the ligand triple (one axial and two equatorial ligands, e.g., ligands 1, 3, 4 and ligands 2, 3, 5 in (**A**) and (**F**) representing the first (**A**–**E**) and second (**F**–**J**) 60° relative rotation/twist steps, respectively), the axial ligand moves to the original position of that equatorial ligand of the ligand triple that will remain equatorial in the new conformation. It should be noted that the sequential steps are provided here only for the sake of clarity and understanding; in reality, these steps occur synchronously and in a concerted manner at the catalytic site in a single-step pathway (see text in Section 2.2.6).

The importance of attempting to derive symmetry rules for flexible skeletons as opposed to purely rigid organic molecules appears to have been first pointed out by Longuet-Higgins [107]. Muetterties subsequently classified some possible mechanisms of the permutational re-arrangements of trigonal–bipyramidal pentacoordinate molecules by regular processes [108]. These concepts were further developed by Hoffmann and colleagues [8]. Berry’s pseudorotation mechanism [109] is the only other viable alternative based on the Muetterties classification. Our intramolecular exchange mechanism is essentially a relative internal rotation mechanism that is similar to the turnstile mechanisms in phosphorus chemistry, and involves twisting/torsional motions by permutation processes in which all ligands participate. The pseudorotation model [109] is different from our proposed mechanism. As its name suggests, pseudorotation involves ligand exchange without an internal rotation or twist of ligands. Besides this, it is a partitioning of the (1–4) ligand set and not a permutation of the (2–3) ligand set involving a pair and a trio of ligands, as proposed here (Figure 6).

Such ligand permutation has not been proposed for F_1_ (except within the torsional mechanism [57,79]). The observation of the oxygen exchange reactions nonetheless proves for a fact that ligands have indeed swapped places, i.e., ligand permutation has occurred. However, it has very recently been proposed for other enzyme systems [112]. The above view is also consistent with the finding in the muscle myosins that the replacement of an oxygen by a sulfur atom in the γ-phosphoryl group, as in the slowly-hydrolyzable ATP analog ATPγS, makes the skeleton more rigid, and abolishes the ligand permutation and the oxygen exchange [110]. It provides a further step into our understanding of why nature chose phosphates [113,114].

The presence of a significant Pi–HOH exchange may also be correlated to the requirement of Pi activation. The Pi activation could then be seen as the tight specific and competent binding of Pi to the catalytic site in the proper conformation for nucleophilic attack (i.e., from MgADP.Pi in L to MgATP in T, as shown in Figure 2 of Ref. [31]).

The presence of a non-variable, exactly 120° external γ-ε rotation in the F_1_ portion of ATP synthase (even in the face of the inter-species variability in the rotational angle per elementary step and the presence of symmetry mismatch in the F_O_ portion [31]) makes it readily visualizable how an overall internal relative rotation (twist) of 120° of two sets of ligands (e.g., a pair and a triple) can occur in the β-catalytic site upon rotation of the γ-subunit and release of torsional energy during ATP synthesis. In particular, we predict that the structurally loose β_TP_ site (L) of F_1_ containing bound MgADP.P_i_ changes its conformation to the structurally tight β_DP-like_ site (T) upon rotation of the γ- and ε-subunits, the removal of γ–β_TP_ interactions, and the formation of key ε–β_TP_ interactions. This occurs especially through the interaction of the helix tip ending in Met–138 of the ε helix–turn–helix motif that funnels ~18 kJ/mol of the released torsional energy stored in γ to the β_TP_ catalytic site. The torsional energy released is instrumental in carrying out the synthesis reaction by forcing the bound, activated P_i_ to crash into the bound MgADP upon converting β_TP_ (L) to β_DP-like_ (T, containing bound MgATP) upon rotation of the top of the γ-subunit. The above molecular mechanism requires energy input at the chemical step of condensation of MgADP and P_i_, and therefore contradicts Boyer’s binding change mechanism and goes beyond it.

Further, the inherent nature of the trigonal bipyramidal reaction intermediates makes for two apical bonds that may be considered to be relatively long, weak, more ionic, more labile and reactive. They are also ideal for harboring the entering or leaving groups. The three equatorial bonds, on the other hand, are shorter, stronger, more stable and non-reactive. For the occurrence of ATP synthesis, it is essential that the bulky ADP moiety be transferred from the apical position to the equatorial and energetically unfavorable position by the ligand permutation process at an intermediate stage of the mechanism (see Figure 5, which pictorially illustrates this equatorial capture of ADP during the process of ATP synthesis). The free energy required for this process would be obtained from the torsional energy stored in the γ-subunit, which is ultimately derived from the energy of the anion and proton electrochemical gradients (Δμ̃_A_^−^ + Δμ̃_H_^+^) [11,15,24,27,36,102,115,116,117,118,119]. In the process of ATP hydrolysis, the bulky ADP residue would be permuted from the equatorial to the energetically favored apical position. The free energy transferred to the γ-subunit of the enzyme by this process would be utilized for the translocation of protons and anions against their electrochemical gradients. This transformation would also be in harmony with the local active site remodeling found in biochemical studies of the chemical mechanism by Pedersen and coworkers [103,104]. The transition from tetrahedral to trigonal bipyramid geometry within the catalytic site naturally leads to the presence of two equatorial –OH groups. The occurrence of both the Pi–HOH and ATP–HOH oxygen exchanges (arrowed dashed lines in Figure 5) is also rationalized since the swapping/interchange of positions of the oxygen ligands of the phosphorus atom occurs readily during the ligand permutation process at intermediate stages of the transformations (Figure 5). Such ligand permutation lies at the heart of the oxygen exchange process, and would also be an essential requirement for the formation of the P_γ_–O bond in ATP synthesis that utilizes the mechanical free energy stored in the γ-subunit of the enzyme as torsional energy.

#### 2.2.6. Stereochemical Consequences

The possibility of a 120° intramolecular relative ligand rotation and twist during energy-linked conformational changes at the enzyme catalytic sites, especially given the presence of an external 120° rotation in the ATP synthase per ATP formed, was not afforded serious consideration previously. The oxygen exchange experiments discussed in this paper also point to the possibility suggested above. In fact, all the studies on oxygen exchange by mitochondrial F_1_-ATPase during ATP hydrolysis in ^18^O labeled water have revealed that the four oxygens atoms of P_i_ were equivalent during the time bound inorganic phosphate engaged in a P_i_–HOH oxygen exchange on the enzyme. This barrier-free unimpeded exchange supports proposals of internal ligand rotation, in which all the ligands participate in the sequence of movements (Figure 5 and Figure 6), and no oxygen ligand is steadily liganded to any group or side chain in the catalytic site. It also offers independent evidence against previous proposals in which one ligand acts as a pivot and does not move [120]. Figure 6 depicts a mechanism that incorporates the above concepts and, by a repetition of the transformation steps shown in Figure 5, also displays the inversion of configuration at the γ-phosphorus, consistent with the experimental data [110,111].

The stereochemical reaction mechanism is shown in Figure 6 with the aid of a hexagonal prism to enable easier visualization of the sequence of steps involving 120° relative rotation and twist of a ligand pair vs. ligand triple. Such changes at the site (e.g., orient Pi in the right conformation, achieve the relative internal rotation/deformation, and force the bound, activated Pi to approach the bound MgADP and form the bond) require energy that can be readily delivered to the catalytic site via γ–β and ε–β interactions, i.e., with the single-copy subunits acting as conduits for energy transmission and coupling. In particular, the structurally loose β_TP_ site (L) of F_1_ containing bound MgADP.P_i_ changes its conformation to the structurally tight β_DP-like_ site (T) by the rotation of the γ– and ε–subunits, the removal of γ–β_TP_ interactions, and the formation of ε–β_TP_ interactions (especially through the critical interaction of the helix tip ending in Met–138 of the ε helix–turn–helix that funnels ~18 kJ/mol of the released torsional energy stored in γ to the catalytic site, and helps to carry out the synthesis reaction by converting β_TP_ (L) to β_DP-like_ (T, containing bound MgATP) through this interaction) with the generation of intramolecular twist and tilt in the γ-phosphate portion. Since no reaction intermediates have been detected experimentally, it is always possible to bypass possible trigonal bipyramid intermediates in the mechanism by a flip/switch of ligands in pentacoordinated phosphorus, as shown in the diagram. It is also possible to derive simple rules for the ligand permutation depicted in Figure 6 based on topological and chemical group theoretical concepts (see legend to Figure 6). There may also be other ways to obtain the 120° relative rotation and stereochemical consequence (inversion of configuration at P^γ^), but they will in all probability be permutationally indistinguishable from the scheme shown in Figure 6. In our opinion, one of the greatest advantages of the mechanism depicted in Figure 6 is its speed. Rotation/twist (by 120°), tilt (by ~18°), the permutation and switch of ligands, and inversion at the γ-phosphorus all occur synchronously and simultaneously, i.e., in a concerted manner. We thus have a one-step pathway, and the permitted dynamic symmetry of the trigonal bipyramid skeleton, i.e., the residual dynamic equivalence of the skeletal positions of the trigonal bipyramid skeleton, is realized in a single step.

Hence, such a twist mechanism consistently rationalizes a wide range of experimental data, and embeds them within and links them to phosphorus’ fundamental chemistry and mechanism [8,107,108,109,110,113,114,121,122,123,124,125,126,127].

## 3. Experimental

New oxygen exchange experiments on submitochondrial particles that lack the nucleotide pools of intact mitochondria have been performed, which have the power to provide definitive and important insights on mechanism. Our experiments on particles, the use of H_2_^18^O in oxygen exchange during the process of ATP hydrolysis by F_1_-ATPase, the effects of uncouplers on the Pi–ATP, Pi–HOH, and ATP–HOH exchanges, methods for the separation of Pi, and the mass spectroscopic determination of the ^18^O present in the various Pi isotopomer species released by the F_1_-ATPase during catalysis are described in this section.

### 3.1. Preparation of Submitochondrial Particles

Submitochondrial particles were prepared by sonic disruption from fresh bovine heart mitochondria by the method of Smith and Hansen [128]. Measurements of the Pi–ATP exchange were made by the separation of Pi from the adenine nucleotides by following the procedure of Martin and Doty [129] for the extraction of the phosphomolybdate complex. Water containing either 0.7 or 10 atom% excess ^18^O was used in the experiments on particles (Sigma-Aldrich, St. Louis, MO, USA). The ^18^O in ATP was determined by nucleotide adsorption on charcoal, followed thereafter by acid hydrolysis. The total extent of the exchange reactions was determined, as discussed below.

### 3.2. Determination of Incorporation of the ^18^O

The total extent of the Pi–ATP exchange reaction was estimated by the measurement of the isotopic fraction, F, by measurement of the specific activity of the acid-labile nucleotide phosphate using the equation

(8) $$Total\;exchange=\left(\frac{\left[Pi\right][ATP]}{\left[Pi\right]+[ATP]}\right)\times F$$

In Equation (8), $\left[Pi\right]$ and [ATP] represent the average pool sizes of Pi and acid-labile nucleotide phosphate, respectively. The latter quantifies the total acid-labile phosphate present, i.e., the β- and γ-phosphoryl group of ATP and the β-phosphoryl group of ADP present. The total extent of exchange, i.e., the atoms of oxygen incorporated from H_2_^18^O into the Pi released into the medium and nucleotide phosphate, is estimated by Equation (9), i.e.,

(9) Atoms of O18 incorporated=4×P×YZ

In Equation (9), P is the average pool size of phosphate during the incubations, Y is the measured atom% excess ^18^O in the phosphate pool, while Z is the atom% excess of ^18^O in medium H_2_^18^O. As already explained, we corrected for 1 oxygen atom incorporated into each molecule of Pi released into the medium upon ATP hydrolysis (Equation (1)).

### 3.3. Oxygen Exchange

In the experiments containing 0.7 atom% excess ^18^O in medium H_2_^18^O, 3 mg of washed bovine heart submitochondrial particles were incubated in a final volume of 5 mL for 15 min at pH 7.5 and 23 °C. The reaction mixture contained 250 mM sucrose, 50 mM Tris sulphate, 10 mM potassium phosphate-^32^P, 10 mM sodium succinate, 1 mM KCN, 1 mM NAD^+^ (Sigma-Aldrich, St. Louis, MO, USA), 40 mM phosphoenolpyruvate (Millipore Sigma, Burlington, MA, USA), 0.01 mg/mL of added pyruvate kinase (Thermo Fisher Scientific, Waltham, MA, USA), and 5 mM Mg^2+^ and varying concentrations of ATP (Carl Roth GmbH, Karlsruhe, Germany). Incubations without added pyruvate kinase were also carried out, and the per cent exchange taking place was quantified with respect to the exchange in the absence of added pyruvate kinase (=100%). The reaction was started by the addition of particles and quenched by the addition of perchloric acid at 0 °C, followed by centrifugation. ATP hydrolysis and the extent of the Pi–ATP, Pi–HOH, and ATP–HOH exchanges were measured in the supernatant as described above. The values plotted are differences between the incubation samples and zero time control samples in which HClO_4_ was added before the particles.

In general, under the above conditions and when respiratory activity is blocked, ATP is rapidly hydrolyzed, and a mixture of ADP and ATP is present. However, the addition of phosphoenolpyruvate and excess pyruvate kinase ensures that ADP concentrations are maintained at a low level.

An experiment with a 10-fold-shorter incubation time (for 90 s) using 10 atom% excess ^18^O in medium H_2_^18^O (Sigma-Aldrich, St. Louis, MO, USA) was carried out similarly at pH 7.5 and 23 °C with 1 mM ATP and 10 mM Pi containing 10^6^ to 10^7^ cpm of ^32^P and 5 mM Mg^2+^. The reaction mixture contained 250 mM sucrose, 10 mM Tris, 10 mM succinate, 20 mM phosphoenolpyruvate, 0.05 mg/mL pyruvate kinase and other additions as given above. These experiments were performed mainly to quantify the relative rates of the three isotope exchanges under low ATP turnover conditions. All the reactions were compared under identical conditions on the same reaction mixture.

### 3.4. Experiments on Particles in the Presence of Uncoupler

The effects of the 2,4-dinitrophenol uncoupler on the inhibition of the three exchange reactions in submitochondrial particles were assessed under the following conditions: 5 mg particles were incubated for 5 min at pH 7.5, 28 °C. The final volume measured 2 mL. The incubation mixture contained 250 mM sucrose, 10 mM Tris-acetate, 10 mM potassium phosphate-^32^P, 0.5 mM ADP, 5 mM ATP, 5 mM MgSO_4_, 0.7 atom% excess ^18^O in medium H_2_^18^O, and varying concentrations of 2,4-dinitrophenol (Sigma-Aldrich, St. Louis, MO, USA).

### 3.5. Separation of Pi

MF_1_-ATPase was isolated using standard procedures [130]. ATP hydrolysis and oxygen exchange experiments on F_1_-ATPase were performed as already described previously in detail [91]. The Pi formed by the F_1_-ATPase was isolated by taking aliquots of quenched reaction mixtures adjusted to 1 mL with H_2_O. Then, 0.5 mL phenol was added and centrifuged to remove the protein with the phenol. The phenol layer was washed with 1 mL of H_2_O, which was added to the original aqueous layer. The phenol present in the combined aqueous layers was extracted into 2 mL of 1:1 isobutenol:benzene by volume. Molybdate was added, the phosphomolybdate was extracted with isobutenol:benzene (Sigma-Aldrich, St. Louis, MO, USA), and the Pi was extracted into an alkaline H_2_O solution. The Pi was separated from the molybdate and converted to H_3_PO_4_ using column chromatography techniques [131]. The H_3_PO_4_ was lyophilized, converted to triethylphosphate with diazoethane, and analyzed for [^18^O]Pi species using GC-MS (Agilent Technologies, Santa Clara, CA, USA).

### 3.6. Mass Spectroscopic Experiments for Determination of ^18^O in Pi

Lyophilized samples of H_3_PO_4_ were derivatized with diazoethane in ethane [131], the excess organic content was evaporated, and the resulting triethylphosphate was dissolved in dichloromethane (Sigma-Aldrich, St. Louis, MO, USA) at a concentration of 1 nmol µL^−1^. Aliquots containing about 1 nmol of sample were analyzed for ^18^O with a Hewlett-Packard GC-MS system. With the injection port at 250 °C, isothermal elution at 160 °C, and a gas flow rate of 30 mL min^−1^, the triethylphosphate was eluted in around 60 s. The diethylphosphate ion fragment of m/z=155 and its corresponding ^18^O isotopomer counterparts characterized by m/z ratios of 157, 159, 161, and 163 were analyzed by selective ion monitoring. These species were designated as ^18^O_0_, ^18^O_1_, ^18^O_2_, as well as ^18^O_3_ and ^18^O_4_ Pi, respectively [57,91].

## 4. Conclusions

An integration of the mechanism of ATP synthesis/hydrolysis within the fundamental framework of phosphorus chemistry has been achieved by exploring the dynamical characteristics of phosphorus–oxygen ligands of ATP molecules in the active site of the ATP synthase/F_1_-ATPase enzyme during catalysis. Fast mechanisms that realize rotation/twist (by 120°), tilt (by ~18°), permutation and switch of ligands, and inversion at the γ-phosphorus synchronously and simultaneously and in a concerted manner have been formulated. Stereochemical consequences of the mechanism have been discussed. These novel insights were made possible by monitoring the oxygen exchange reactions accompanying ATP synthesis/hydrolysis occurring in the active sites of β-subunits at the α–β interfaces of the F_O_F_1_-ATP synthase/F_1_-ATPase during its enzymatic cycle. These considerations lead to new concepts of ligand permutation about the phosphorus center for the rationalization of the oxygen exchange reactions during catalysis of ATP synthesis/hydrolysis. Based on experiments reported on submitochondrial particles in this work, new kinetic data on the relative rates of the intermediate Pi–HOH, ATP–HOH, and Pi–ATP oxygen exchange reactions, the inhibition of the exchanges upon ADP removal in the presence of an ATP-regenerating system, and uncoupler action have been developed in detail. The experimental results reveal the inability of excess pyruvate kinase and phosphoenolpyruvate, and thereby of ADP removal, to inhibit the intermediate Pi–HOH exchange when ATP and submitochondrial particles were incubated, and show that the nucleotide requirement of the intermediate Pi–HOH exchange is sufficiently met by ATP, but not by ADP.

A new scheme that better clarifies the mechanistic origin of oxygen exchange was needed to explain the kinetic results. The results suggest that ligand permutation is the fundamental and root cause of the occurrence of the prominent intermediate Pi–HOH exchange (during ATP hydrolysis) and the rapid intermediate ATP–HOH oxygen exchange (during ATP synthesis) relative to the Pi–ATP exchange, as opposed to multiple spontaneous reversals of ATP synthesis/hydrolysis in a single catalytic site. The exchanges were concluded to occur mechanistically because the enzyme catalytic site lacks absolute spatial selectivity for the oxygen ligands of a phosphorus intermediate that it accepts and binds as substrate, and that the oxygen ligands readily exchange, i.e., permute their positions about the central phosphorus atom.

It was concluded that the results of our study contradict the central postulate in Boyer’s binding change mechanism of reversible catalysis at a F_1_ catalytic site with $K_{eq}$ close to unity [33,34,59,60] that predicts an absolute requirement of ADP for the occurrence of the Pi–HOH exchange [81]. The prominent intermediate Pi–HOH exchange occurring under ATP hydrolytic conditions has been shown to be best explained by Nath’s torsional mechanism of energy transduction and ATP synthesis/hydrolysis, which made its first appearance 25 years ago [62,63,64], and postulates an essentially irreversible cleavage of ATP by mitochondria/particles [33], without a reversible formation of ATP from ADP and Pi at the catalytic site. The explanation within the torsional mechanism has also been shown to rationalize the relative insensitivity of the intermediate Pi–HOH exchange to uncouplers observed in the experiments compared to the intermediate ATP–HOH and Pi–ATP exchanges. These developments offer exciting new perspectives on the mechanism of ATP synthesis and hydrolysis based on the fundamentals of phosphorus chemistry, and take us well beyond the binding change mechanism of ATP synthesis/hydrolysis in bioenergetics.

## Data Availability

The data are contained within the article.
